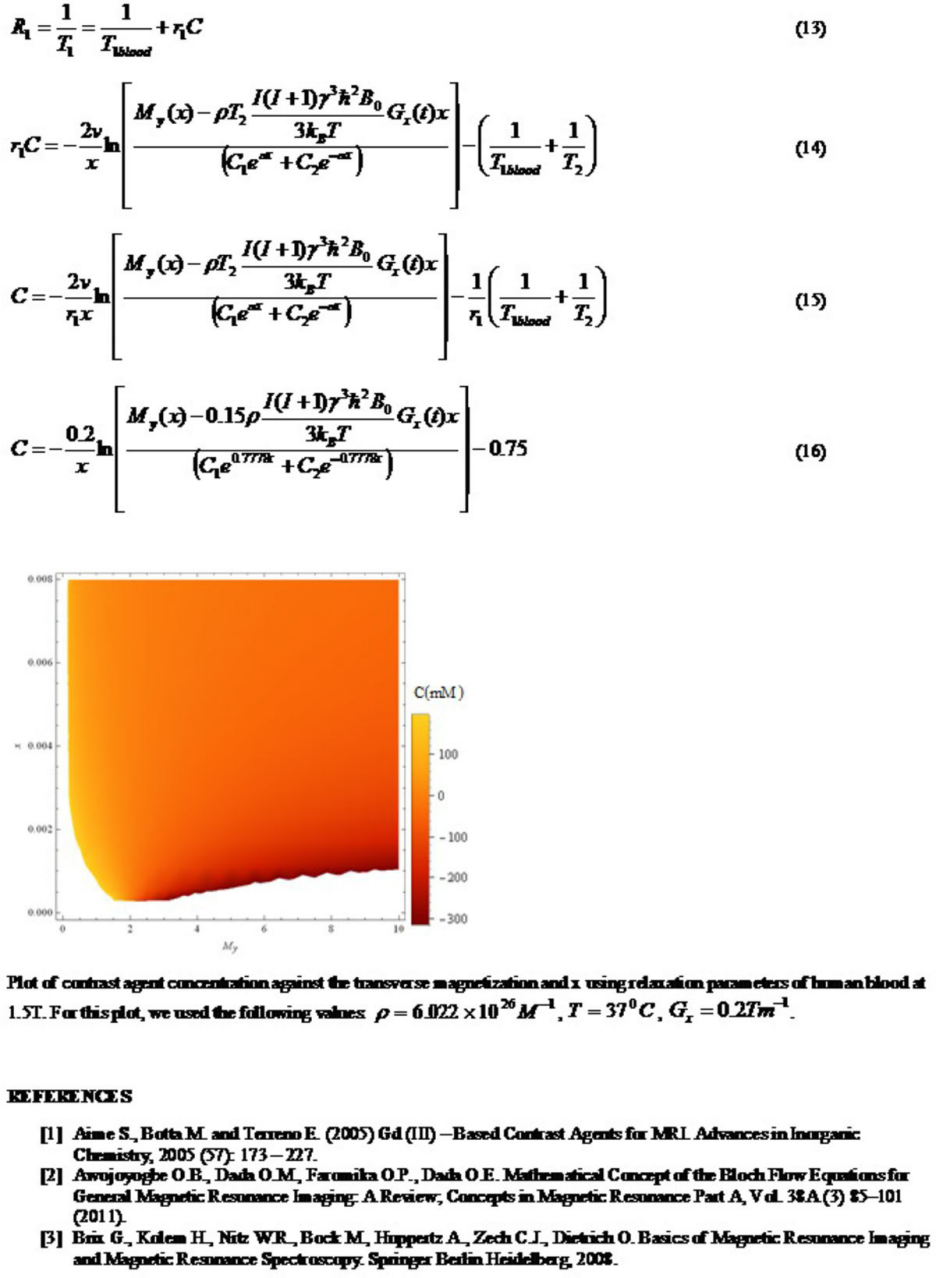# Computational model of bloch flow equation for in-vivo assessment of contrast agents

**DOI:** 10.1186/1532-429X-16-S1-O93

**Published:** 2014-01-16

**Authors:** Michael O Dada, Simona Baroni, Bamidele O Awojoyogbe

**Affiliations:** 1 Physics, Federal University of Technology, Minna, Niger State, Nigeria; 2 Chemistry, Molecular Biotechnology Centre, Via Nizza 52, Turin, Piedmont, Province of Turin, Italy

## Background

Responsive contrast agents (CA) are diagnostic agents whose contrasting features are sensitive to a given physico-chemical variable that characterizes the microenvironment of the probe[1]. The crucial diagnostic parameters are pH, temperature, enzyme activity, redox potential and concentration of specific ions, and low weight metabolites[1]. However, their clinical use is still far-off because accurate measurement of the parameters with a given probe requires a precise knowledge of the local concentration of the contrast medium in the region of interest. If this can be achieved, we can then safely attribute observed changes in relaxation rate to changes in the parameters to be measured[1]. Although novel classes of MRI paramagnetic CAs which address this problem have been successfully developed[1], a possibility exists that the challenges above may actually be a blessing in disguise; ie, changes in the local concentration of the CA in the region of interest may serve as additional source tissue contrast but provided we can infer the local concentration changes directly from measures MRI signal.

## Methods

In this study, we shall attempt to develop a computational model which relates the measured MRI signal in terms of the M_y _to the tissue temperature and concentration of the CAs. Such model may be tested in-vitro for the purpose of setting a reference point for parameter relationship and then used to predict CA dynamics in-vivo. The time - independent Bloch flow equation is[2] eqn (1). For this study, we assume that Larmor condition (egn (2a)) holds and within the small RF limit, eqn (1) becomes eqn (2b). For paramagnetic complexes, the Curie law[3] is eqn (3). The applied RF field can be related to the gradient field and given as eqn (4). The complimentary function is given as eqn (6). Due to the nature of the right hand side of eqn (5), we shall assume a solution for the particular solution as eqn (7). Substituting for this expression in eqn (5) gives eqn (8). Equating the coefficients of like powers of x, we have eqn (9). Therefore, the general solution is given in eqn (10).

## Results

In contrast - enhanced MRA, the reduction of T_1 _after administration of a CA is used. When a CA is injected, the T_1 _of blood is shortened from T_1 blood _= 1.2 s (at B_0 _= 1.5T) to less than 100 ms during the first bolus passage [3]. The relaxation rate as a function of the local CA concentration (C) is given [2, 3] in eqn (13). If the CA does not have any significant influence on T_2_, we have eqn (14). Using the values [1, 3]: T_1 blood _= 1.2 s, T_2 _= 0.15 s, r_1 _= 10 mM^-1^s^-1^, v = 1.0 m/s (aortic arch), we have eqn (16). With the expression in equation (16), we can now easily correlate changes in C to the observed MRI signal, CA properties and imaging parameters.

## Conclusions

This computational model can be used to image changes in the local concentration of CAs in-vivo. Once reference points have been set in - vitro, we can easily infer parameter changes from the measured MRI signal.

## Funding

None.

**Figure 1:**
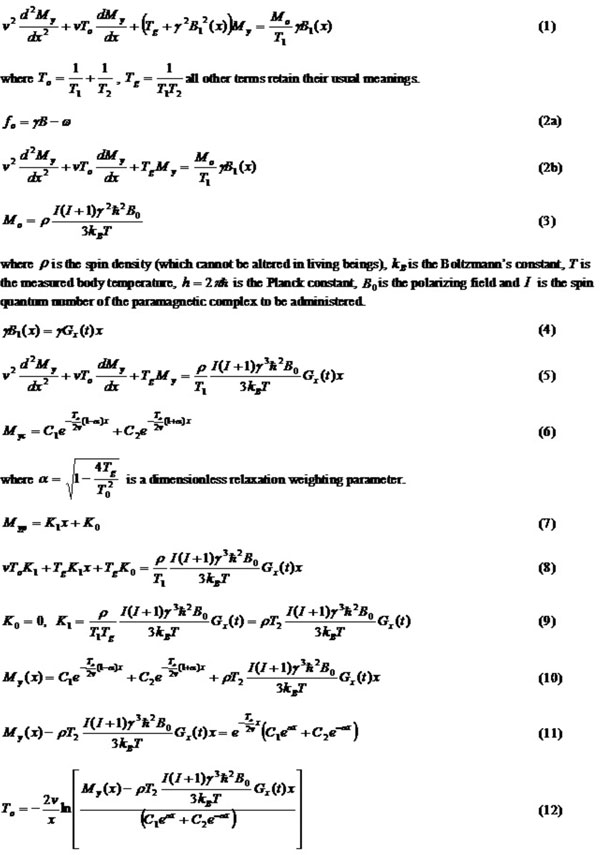


**Figure 2:**